# Sports ingroup love does not make me like the sponsor’s beverage but gets me buying it

**DOI:** 10.1371/journal.pone.0254940

**Published:** 2021-07-28

**Authors:** Sara Franco, Ana Maria Abreu, Rui Biscaia, Sandra Gama

**Affiliations:** 1 Department of Computer Engineering, Instituto Superior Técnico, Universidade de Lisboa, Lisbon, Portugal; 2 Center for Interdisciplinary Research in Health, Institute of Health Sciences, Universidade Católica Portuguesa, Lisbon, Portugal; 3 Department for Health, Faculty of Humanities and Social Sciences, University of Bath, Bath, United Kingdom; 4 Group of Graphics and Interaction, INESC-ID, Lisbon, Portugal; Iowa State University, UNITED STATES

## Abstract

Previous literature has shown that social identity influences consumer decision-making towards branded products. However, its influence on ones’ own sensory perception of an ingroup (or outgroup) associated brand’s product (i.e. sponsor) is seldom documented and little understood. Here, we investigate the impact of social identity (i.e. team identification) with a football team on the sensorial experience and willingness to buy a beverage, said to be sponsoring the ingroup or the outgroup team. Ninety subjects participated in one of three sensorial experience conditions (matched identity: ingroup beverage; mismatched identity: outgroup beverage; control: no group preference). Each participant tasted the new sponsoring beverage and answered a questionnaire about their subjective sensorial experience of the beverage. EEG and BVP were synchronously collected throughout. Analyses revealed that team identification does not influence subjective responses and only slightly modulates physiological signals. All participants reported high valence and arousal values while physiological signals consistently translated negative affects across groups, which showed that participants reported to be happy/excited about trying the beverage while their physiological signals showed that they were feeling sad/depressed/angry. Crucially, despite a similar sensorial experience, and similar socially desirable report of the subjective experience, only participants in the matched identity group demonstrate higher willingness to buy, showing that the level of team identification, but not taste or beverage quality, influences willingness to buy the said sponsor’s product.

## Introduction

Many studies have investigated the impact of team identification in sports consumer habits [1–10]. Team identification is defined as the degree of psychological connection a fan has with a sport team [1], and commonly used as a measure of attitudinal loyalty towards the teams [2]. For many sports fans, identifying with a team presents the psychological benefit of a sense of belonging (cognitive realization of a connection to a team) and, thus, an enhanced sense of self [3, 4], which often translates into positive attitudinal and behavioural outcomes for sport entities [3, 5] and associated sponsors [2, 6]. One of most desirable outcomes for sponsors is increased purchase intentions [6] given that it serves as a proxy for fans’ actual purchase behaviours [6]. Purchase intentions refer to the person’s conscious plan in exerting an effort to purchase a brand [7]. Fans may buy the sponsors’ products as an extension of goodwill or to repay the sponsor for supporting the team [8]. The stronger the link with the team, the more fans might feel it is their duty to purchase the sponsors products [9].

Social identity theory [11, 12] represents a solid background for understanding team identification [3], which posits that individuals derive a greater sense of self from the perceived awareness, value, and emotional significance of belonging to a group [11]. Individuals seek to maintain a positive social identity, which derives from favourable comparisons between an ingroup and outgroup [10]. Thus, an individual’s acknowledgement of a group’s existence requires at least one other group, distinct from the ingroup to which he/she belongs. Previous research found that sports fans often show ingroup favouritism and outgroup derogation, and that these biases often occur when subjects have a higher identification with the team in question [1]. In addition, researchers investigated if the bias effect would be most pronounced in situations involving threat to one’s social identity [13], and verified that the individual’s psychological connection to his/her team played a vital role in the level of bias, since the greatest level of bias was reported by highly identified fans.

Also, according to the social identity theory, a salient social identity can determine cognitive focus as well as affective and behavioural responses [14]. However, it remains to be investigated if ingroup/outgroup identity might influence the low-level sensorial experience of a product. Here, we aimed to address this issue in order to verify if the decision concerning the sensorial qualities of a beverage and purchase intentions, is associated with a sensorial self-reported subjective experience (Bottom-Up mechanism) or if there is an heuristic that affects the sensorial experience (Top-Down mechanism). Top down mechanisms are possible contenders for modulating these decisions, since heuristics are cognitive techniques that act as shortcuts to facilitate problem solving and simplify decision-making during situations of uncertainty [15] but when applied indiscriminately can lead to cognitive bias and suboptimal decision-making [16, 17]. Indeed, in a top-down mechanism, people’s perceptions result from mental constructions in which contextual effects (previous experience and knowledge, expectations and/or emotional state) interfere with how the input is processed and evaluated, and how the final decision is derived [18–20]. On the other hand, in a bottom-up mechanism the information flows in a feed-forward fashion—from the sensory stimulus itself, through perceptual analysis, towards the motor output [18]. We used physiological signal analysis and subjective affective responses using the Self-Assessment Manikin (SAM) [21], in order to obtain and compare bottom-up with top-down valence and arousal values. Hence, if a bottom-up mechanism takes precedence, then, the physiological data should match the self-reported answers (in SAM), as it is the sensory stimulus that informs decision making. Contrarily, a top-down mechanism might be present if the self-reported answers do not match the physiological data and, in this case, one might infer that team identification may have influenced the participants subjective responses.

In summary, we explore the ingroup/outgroup influence on both subjective and affective sensorial experience of a beverage as well as purchase intentions. Specifically, we explore team identification with a football team, since football has played a key role in shaping and cementing senses of national identity throughout the world [22] and it is the sport with the greatest economic impact in Europe [23].

In light of the aforementioned literature, we expect that participants with higher team identification who taste the ingroup beverage (sponsoring beverage from their own team) will present positive physiological responses (positive valence) as well as positive subjective responses in tandem with purchase intentions greater willingness to buy the sponsor’s beverage [24]. On the other hand, we expect that participants who taste the outgroup beverage (sponsoring beverage from the rival team) will present both negative physiological and subjective responses, along with lower purchase intentions. Moreover, we expect that highly identified fans would present higher physiological arousal levels as well as higher self-reported arousal values [25] regarding the ingroup beverage tasting.

In accordance with what has been described above, the following Hypotheses are proposed:

Hypothesis 1 (H1): Ingroup identity leads to positive and aroused emotional states while outgroup identity leads to negative and idled emotional states.
Hypothesis 2 (H2): Ingroup identity leads to positive subjective reports of sensorial experience while outgroup identity leads to negative and idled subjective reports of sensorial experience.
Hypothesis 3 (H3): Ingroup identity leads to higher purchase intentions while outgroup identity leads to lower purchase intentions.

## Methods

### Participants

The participants’ identification with the team was measured through an online survey spread throughout different social media platforms and football fan groups. The survey was answered by 497 subjects, from where we selected 104 subjects non-affiliated with any football team, 129 affiliated with team A and 153 affiliated with team B to further recruit through email. Finally, we selected a total of 92 participants with a higher composite measure of team identification for A and B groups, and lower composite measure of team identification for the Control group. Since team identification represents a psychological connection to the team which is intensified by favourable comparisons between ingroups and outgroups (i.e., highlighting the goodness of the ingroup) [10], and it is manifested through several types of behaviours, including game attendance and owning a membership card [3, 26], more than simply measuring the identification with a team, we also considered the behaviours related to that identification, thus creating this composite score. In order to obtain this composite measure of team identification, we used a weighted sum of the answers to the queries related to: interest in football (*football*_*interest*) (0–10); team support (*supporter*) (0–10); game attendance (*game*_*attendance*) (0–10); opposition to the rival team (*opponent*) (0–10); and ownership of a membership card (*membership*_*card*) (0 for the absence of membership card and 1 for card ownership). The former 4 factors were assessed via a VAS scale, while the latter was achieved by direct attribution of points in accordance to the ownership situation. Thus, the team identification score (*identity*_*score*) was calculated using Eq 1, ranging from 19 to 45 for matched and mismatched identity groups. For inclusion in these groups, we considered a minimum score of 19, corresponding to an interest in football of at least 7 or more (out of 10), support for team A or B of 7 or more (out of 10) and game attendance of at least fifty percent of the home games (5 out of 10) (e.g. 7+7+5 = 19). For the control group we selected participants with no football interest, and whom do not support any football team, so they were automatically scored with zero. The actual questions participants answered to regarding their team identification are provided in the S1 Appendix.

$$\begin{array}{c} \begin{array}{cc} identity\_score & =football\_interest+supporter+\\ & game\_attendance+opponent+5*membership\_card \end{array} \end{array}$$ (1)

A total of 92 participants were recruited. Due to equipment malfunctions or technical problems, two participants were discarded. We tested three independent groups of 30 participants: A identity, B identity and Control (25 men and 5 women in each group). The 90 participants were all Portuguese and ranged in age from 18 to 60 years with the A group (M = 30.40, SD = 11.75), the B group (M = 27.57, SD = 11.37) and the Control group (M = 27.27, SD = 11.41), presenting no significant differences in age distribution (*p* = 0.518). Testing was spread throughout the months of October 2020 to January 2021 due to the pandemic constraints. Inclusion criteria implied that participants would not present any type of allergy related with carbonated beverages that might affect the results of the task. Information concerning the experimental hypothesis was only shared after completion of the experimental sessions by all participants in order to avoid self-report and other biases. Participants were assigned with a unique identifier that was used for drawing one gift card worth 20EUR.

### Apparatus & measures

**Fig 1:**
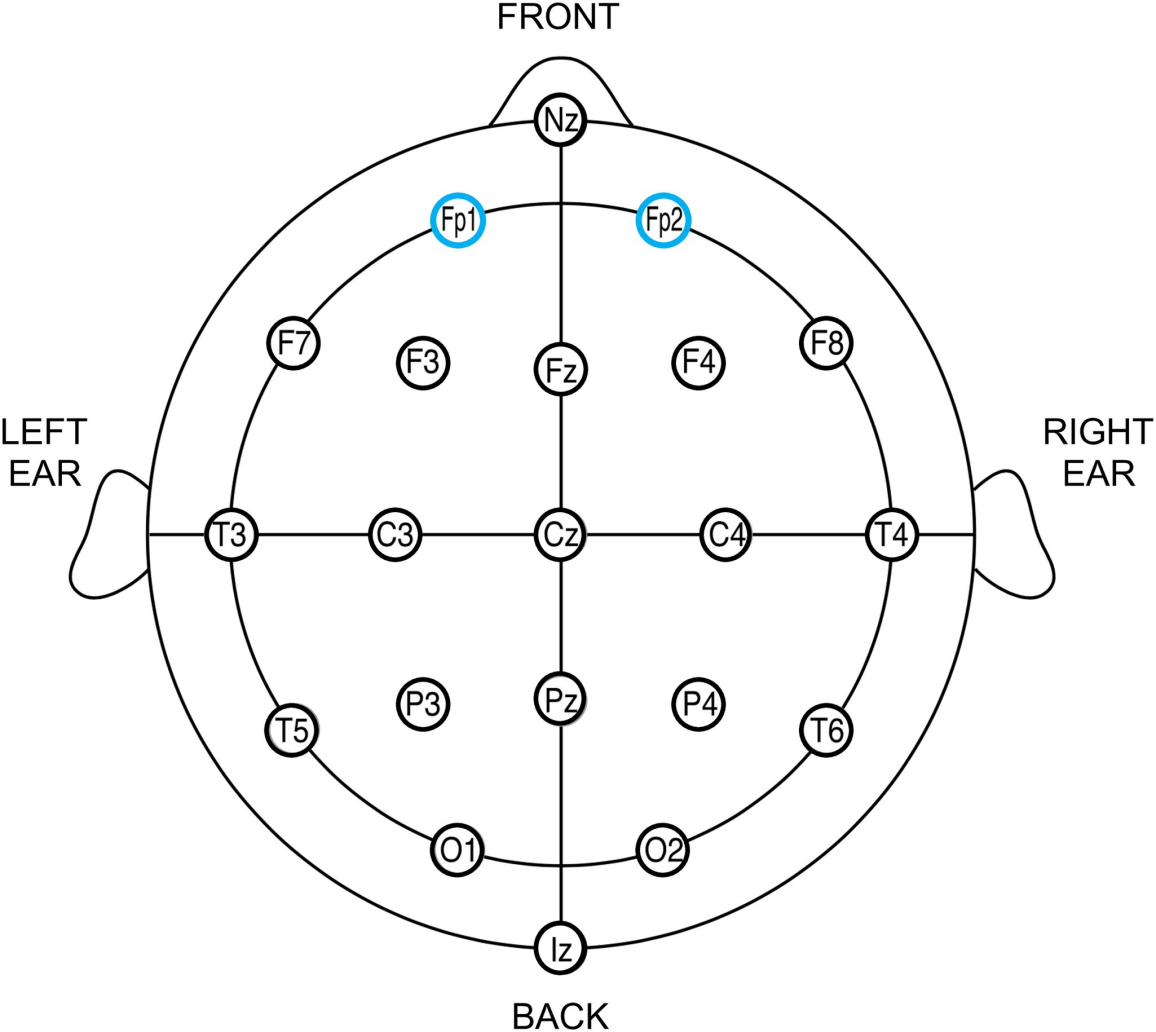
10–20 system.

Physiological data was recorded at 1000 Hz with OpenSignals (r)evolution Mac OS Catalina (v.2019) software available in www.bitalino.com/en/software. This software is used for real-time biosignals recording and visualization, adapted to interact directly with BITalino, which we used in this study to measure physiological data. We used a BITalino to record the Blood Volume Pulse (BVP) that allowed instantaneous Heart Rate (HR) assessment and the Electroencephalography (EEG) that allowed the collection of information concerning brain activity, specifically alpha bands, using three electrodes placed on the forehead (positions FP1 and FP2 in the 10–20 system [27], as presented in Fig 1) and one electrode near the ear on the mastoid (to serve as “ground” for the difference in both hemispheres). The BVP sensor was placed on the forefinger on the opposite hand the subject used to write, since the physiological signals continued to be collected while filling out the questionnaire. We collected EEG and BVP since EEG has been used to investigate the potential of monitoring emotional valence [28–30], in particular the alpha (*α*) wave, while BVP has been commonly used as an indicator of psychological arousal in affective computing [31].

As previously stated, in order to assess the self-reported subjective sensorial experience, we used the SAM pictographic scale. The SAM is a non-verbal method for quickly assessing valence and arousal associated with an individual’s emotional reaction to an event. It is a nine-point scale, where the most leftward (most unpleasant and calm) and the most rightward (pleasant and aroused) parts of the scale corresponded to values of 1 and 9, respectively. The SAM has been widely used to assess subjective emotional response [32], since it allows the measurement of affective valence and arousal while making use of a more human-like figure for reliable decisions on perceived emotion [33]. This scale has also been used to assess sensorial experiences such as beverage tasting [34]. Furthermore, the SAM scale was found to be a reliable measure of emotion that is strongly correlated with peripheral physiological measures, which were used in our study [35–37]. Originally, this scale is composed by three dimensions: valence, arousal and dominance. However, we did not include the dominance dimension since we aimed to make a correspondence between the physiological signals and the self-reported measures. Since, we would be able to address the dominance dimension with physiological measures, we would only obtain a subjective measure. Furthermore, previous studies [38, 39] demonstrated that dominance does not significantly influence consumer behaviour. Thus, by not including we aimed to keep a clearer rationale and avoiding overcrowding the reader with additional hypotheses. The actual questions the participants answered to regarding the self-reported valence and arousal are presented in the S1 Appendix.

Moreover, we used three questions to investigate purchase intentions [40]. Two questions were answered using a 10cm vertical VAS scale and one question was a multiple choice. The VAS was used in conformity with previous studies [41, 42]. This scale has been used to assess subjective experiences and allows to measure more accurately what people are actually experiencing [43]. The questions are presented in the S1 Appendix.

Moreover, we selected six images from the International Affective Picture System (IAPS) [44]. IAPS is a picture database for studying emotion and attention, widely used in research. The images selected for this study contained landscapes, sunsets, flowers and families, since previous studies have established that these images lead to low values of arousal and high levels of valence, which correspond to a relaxing state [45, 46]. Furthermore, these stimuli have been used in the affective research for many reasons, one of them because it is a measure that is based on a dimensional theoretical account of emotions [21, 47, 48], which is of great interest to our study, since we evaluate the emotional states in terms of valence and arousal dimensions. Therefore, we used these images to induce the same relaxing state in each participant before the beverage tasting, to serve as a baseline and allowing the analysis of HR and EEG *α* variations in each participant. Thus, we were able to assure that the variations pertained physiological changes were associated to the sensorial experience (tasting) and not to previous subjective individual emotional states.

### Procedure

**Fig 2:**
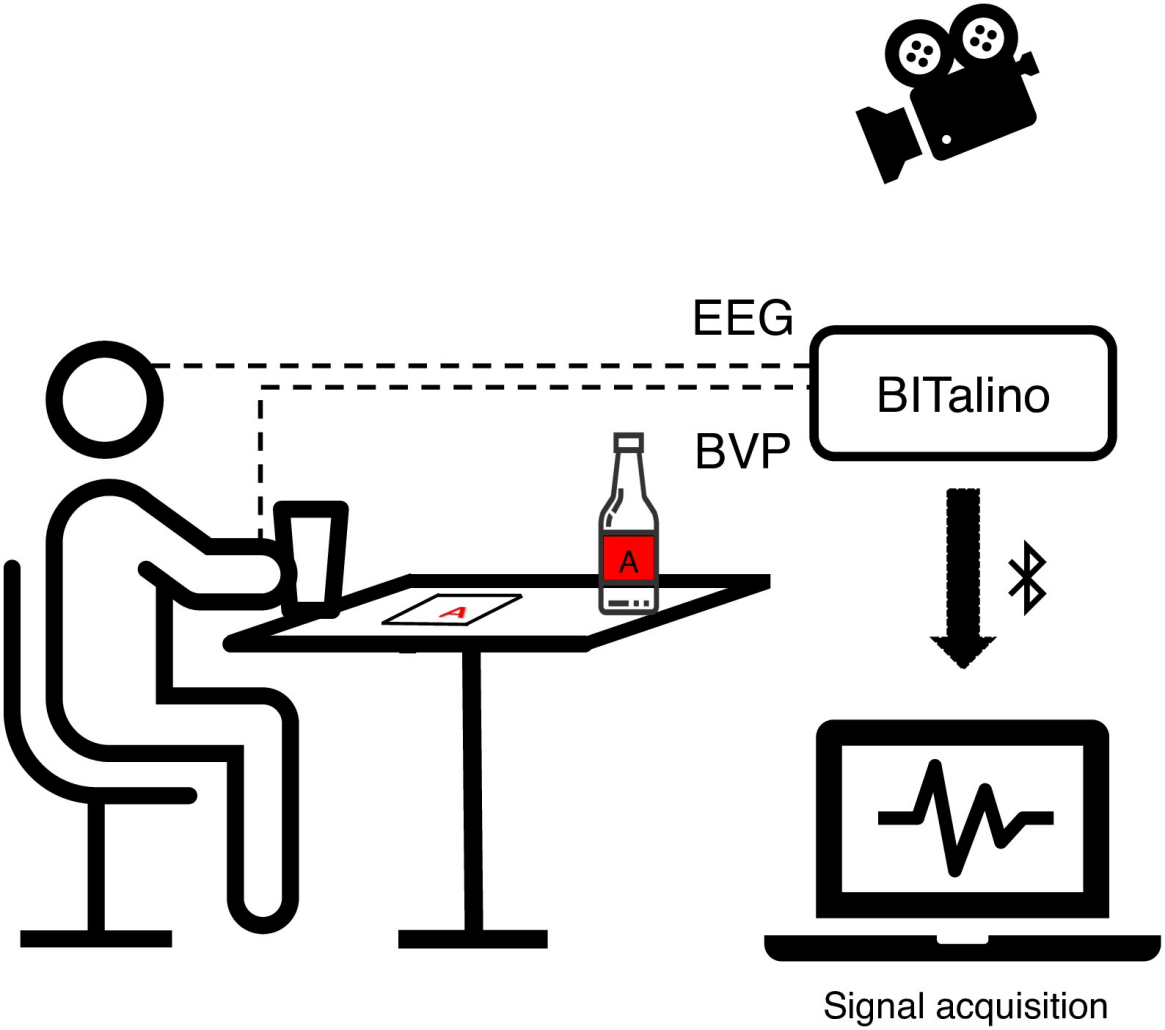
Experimental setup.

**Fig 3:**
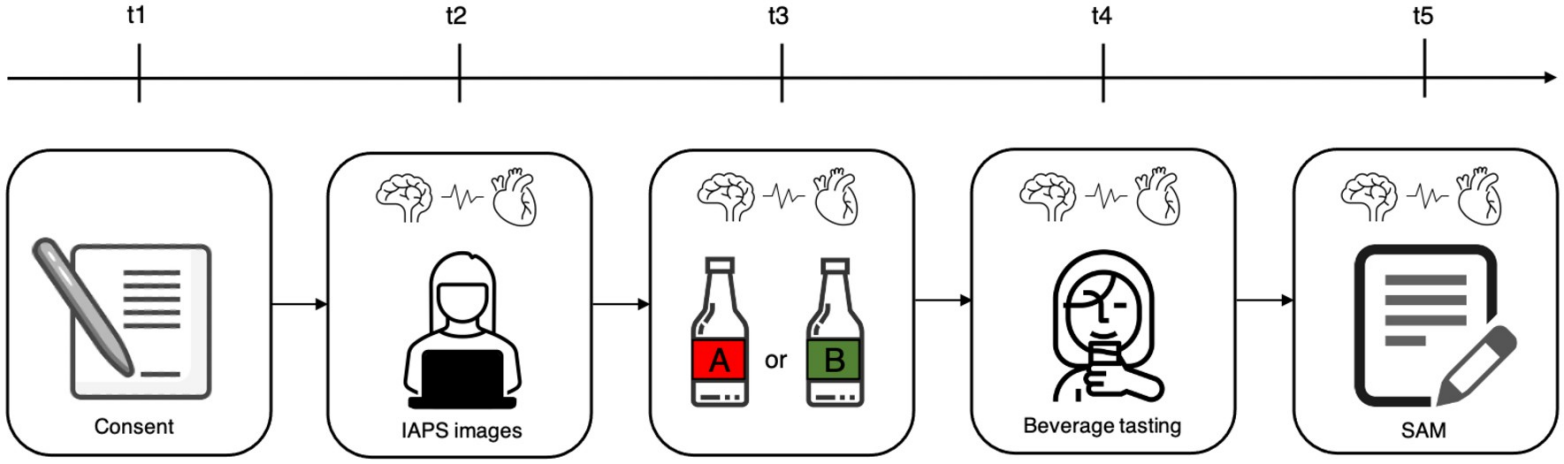
Experimental design timeline.

The experimental study was performed in a controlled environment in a room at a Local Laboratory, in order to minimize the number of distractors and to avoid interference during the experimental session. For experimental setup please see Fig 2. Design timeline is presented in Fig 3. Each participant was tested individually. The COVID-19 protocol implicated that, upon arrival, the participants changed their mask and cleaned their hands with an alcohol-based hand sanitizer. Informed consent (also sent beforehand for previous reading) was obtained from all participants, giving us permission to collect their physiological signals and record the session in video (t1). Participants were requested to sit comfortably on a chair and to look at the chosen set of images from IAPS (presented on the computer screen) for two minutes and then keep their eyes closed for another two minutes, thus following the protocol to achieve a relaxing state [49] (t2).

EEG and BVP were measured with BITalino multi-sensor acquisition platform throughout the session. Participants were instructed to keep their head and the BVP sensor hand as still as possible during the session, in order to prevent detaching the electrodes and capturing undesirable artifacts. We then introduced the new beverage said to be sponsoring team A or B (depending on each sub-group the participant was inserted in), and shared a flyer about the new beverage and presented the bottle prototype (t3). Since colour has been shown to influence the perception of taste [50], we chose a transparent bottle in order to prevent such bias. The beverage chosen for this study was a watermelon flavoured carbonated drink with transparent color, a beverage less identifiable in the Portuguese context. We poured the beverage in a transparent glass for the participants to taste (t4). This process was repeated with six different combinations of ingroup or outgroup identification: 15 participants who identified with team A tasted A’s sponsor beverage (AxA), 15 participants who identified with team A tasted B’s sponsor beverage (AxB), 15 participants who identified with team B tasted B’s sponsor beverage (BxB), 15 participants who identified with team B tasted A’s sponsor beverage (BxA), 15 participants from the Control group tasted team A’s sponsor beverage (ControlxA) and lastly, 15 participants from the Control group tasted team B’s sponsor beverage (ControlxB).

Participants were instructed to answer a series of questions concerning the beverage’s subjective sensorial experience using the SAM pictographic scale (t5). The participants thus reported their subjective responses concerning the overall perceived quality of the beverage as well as four specific sensory attributes of the beverage [51], i.e., the quality of being refreshing [52], thirst-quenching [50], flavoursome [53] and texture quality [53]. This questionnaire also included questions about the beverage’s purchase intentions, measured through VAS.

### Data processing

BITalino measured the analog signals produced by each participant. As mentioned before, these signals (RAW data) were loaded with OpenSignals (r)evolution. After running a script to separate and process the files from OpenSignals with formulas provided in the technical sheets of the BITalino sensors, we used the BioSPPy tool [54], a toolbox for biosignal processing written in Python, in order to process the data, allowing us to obtain the correct values for analysis of the valence and arousal.

In order filter the noise and to increase EEG signal quality, a Butterworth low-pass filter with a cut-off frequency of 40 Hz and a Butterworth high-pass filter with a cut-off frequency of 4 Hz were applied. A Hanning window with 50% overlap was also applied and submitted to Fast Fourier Transform in order to avoid excluding information at the boundary of the windows. Average alpha power in the 8–13 Hz band was taken as an index of alpha power. For the BVP, a finite impulse response band-pass filter with a boxcar smoothing kernel was applied.

The resting state period was defined by the endmost 20 seconds watching the IAPS images together with the seconds wherein the participants had the eyes closed, discarding the last 10 seconds with eyes closed to prevent possible errors. In order to normalise the data, we used the median of the resting state records as a reference to compare each participant’s signals variation, since the effect of outliers in the median is very small or not existent [55], making our processing independent of outliers. Thus, the values used for comparison between each participant were the means of the result of subtracting the values of beverage tasting and the median value of the baseline, allowing for comparisons of instantaneous HR and EEG alpha bands for each participant.

In order to compare and interpret physiological data and subjective responses, we used Min-max normalisation [56] to convert the data. This normalisation technique allowed us to scale arousal and valence values between -4 (*NewMin*) and 4 (*NewMax*) and map the *NewValue*s in a 2D chart wherein the Valence is represented in the X axis and Arousal is represented in the Y axis. To convert the values, the following formulas were used, where *OldMax* and *OldMin* are the maximum and minimum values before conversion, respectively:

$$\begin{array}{c} OldRange=OldMax-OldMin\\ NewRange=NewMax-NewMin\\ NewValue=(((OldValue-OldMin)*NewRange)/OldRange)+NewMin \end{array}$$ (2)

Regarding the purchase intentions, we computed a willingness to buy score (*willingness*_*to*_*buy*) composed by the weighted sum of three features: how much the participant would recommend the beverage to his/her family or friends, how much the participant would like to buy the beverage when it becomes available in the market, both measured on a 10-cm vertical VAS, and how much would the participant be willing to pay for the beverage (not willing to pay = 0; less or equal to 2EUR = 5 and more than 2EUR = 10).

### Statistical analysis

We verified that there were no differences in physiological signals as well as self-reported valence and arousal values between AxA and BxB, between AxB and BxA and also between CxA and CxB, so we clustered these subgroups into 3 more encompassing groups: matched identity—participants who tasted the ingroup beverage (AxA + BxB); mismatched identity—participants who tasted the outgroup beverage (AxB + BxA); and control—participants who did not identify with any football team (CxA + CxB).

Thus, in order to investigate differences in the physiological signals results parameters between the three groups, and since data was not normally distributed, we performed a Kruskal-Wallis H test with the factors Group (matched identity, mismatched identity and control) and Physiological Signals (EEG *α* and HR). Regarding the self-reported responses, we conducted between-subjects ANOVA to examine differences in the self-reported arousal and valence values between groups, with sensory attributes concerning the beverage tasting (overall quality, freshness, thirst-quenching, flavour and texture) as dependent variables. For those attributes where we found significant differences, we further tested multiple comparisons with Tukey’s post hoc test.

In order to evaluate if there was an association between the *identity*_*score* and the subjective valence and arousal responses, we performed Pearson Correlations for both valence and arousal (matched and mismatched identity groups). Further Spearman Correlations were conducted to investigate if there was an association between *identity*_*score* and the physiological responses for the same two groups. We also performed Pearson Correlations between *identity*_*score* and *willingness*_*to*_*buy*, for the matched and the mismatched identity groups as well. Furthermore, we conducted a Kruskall-Wallis H test to investigate putative differences in *willingness*_*to*_*buy* between the three groups. For those where we found statistical differences, we performed Man-Whitney tests between pairs of the independent variables (groups). Statistical results are presented in the Results section.

### Ethical statement

Signed informed consent was obtained from all participants before the experiment. Ethical approval for the study and the protocol for sharing the anonymised data were obtained from the Ethical Committee of Instituto Superior Técnico (http://etica.tecnico.ulisboa.pt/). Behavioural testing and physiological data were collected according to the ethical principles of the Declaration of Helsinki, 2013 [57].

## Results

Before conducting the statistical analysis, we tested both *identity*_*score* and *willingness*_*to*_*buy* for internal consistency by computing the Cronbach alpha scores, using the coefficient of 0.7 as a guideline in the analyses [58]. The results showed that the measurement scales of the *identity*_*score* were consistent and stable for both teams A (Cronbach alpha = 0.762) and B (Cronbach alpha = 0.702). Regarding the purchase intentions, the measurement scales of the *willingness*_*to*_*buy* was also found to be reliable and consistent (Cronbach alpha = 0.727).

### Physiological and subjective responses

We first analyzed the possible differences on physiological responses between subgroups which tasted the ingroup beverage (AxA and BxB), between subgroups which tasted the outgroup beverage (AxB and BxA) and also between subgroups not identified with any football team (CxA and CxB). Thereunto, we conducted Independent Samples t-tests which revealed that there were no significant differences regarding EEG *α* (ALPHA) between AxA (M = 6.68E-14, SD = 8.04E-14) and BxB (M = 1.03E-13, SD = 8.32E-14) subgroups (t(28) = -1.20, p = 0.24), nor between AxB (M = 7.88E-14, SD = 4.25E-14) and BxA (M = 9.53E-14, SD = 1.84E-13) subgroups (t(28) = -0.34, p = 0.74) or between CxA (M = 7.04E-14, SD = 3.37E-14) and CxB (M = 9.07E-14, SD = 5.86E-14) subgroups (t(28) = -1.17, p = 0.25). Likewise, we did not find significant differences regarding HR between AxA (M = 5.72, SD = 8.75) and BxB (M = 7.58, SD = 9.96) subgroups (t(28) = -0.54, p = 0.59), nor between AxB (M = 8.67, SD = 14.54) and BxA (M = 0.81, SD = 17.74) subgroups (t(28) = 1.33, p = 0.20) or between CxA (M = 6.76, SD = 17.02) and CxB (M = 9.34, SD = 9.79) subgroups (t(28) = -0.51, p = 0.61). Independent Samples T-tests were also performed to compare the self-reported valence and arousal values between those subgroups and results showed once more that there were no significant differences between any sensory attribute. So, as stated before, we clustered these subgroups in three groups: matched identity, mismatched identity and control and performed the remaining statistical analysis considering these 3 groups. Table 1 presents the means by group for all variables, providing a quick overview of the results.

**Table 1:** Mean for all measures, by group.

	Mean			Significance
	matched identity	mismatched identity	control	
IDENT	30.47	33.40	0.00	**0.00**
ALPHA	8.47e-14	8.70e-14	8.05e-14	0.96
HR	6.65	4.74	8.05	0.64
OQ_V	6.83	6.40	6.87	0.47
OQ_A	5.93	5.60	5.40	0.37
FRSH_V	7.40	6.43	6.97	**0.04**
FRSH_A	6.73	6.10	5.97	0.13
THRST_V	6.67	5.23	5.57	**0.01**
THRST_A	6.23	5.10	4.93	**0.03**
FLV_V	7.50	6.40	6.80	0.11
FLV_A	6.80	6.40	6.37	0.53
TXT_V	6.80	6.40	5.93	0.14
TXT_A	6.30	5.70	5.67	0.32
PRCH_INT	19.05	12.82	13.33	**0.00**

With the aim of examining differences in physiological values between the matched identity, the mismatched identity and the control groups, we conducted a Kruskall-Wallis H test. No significant differences were found for EEG *α* (*χ*^2^ = 1.61, p = 0.45, df = 2) nor for HR (*χ*^2^ = 0.50, p = 0.78, df = 2). Therefore, hypothesis H1 was not supported. On the other hand, we performed a 3 x 5 between-subjects ANOVA for valence and a 3 x 5 between-subjects ANOVA for arousal to investigate possible differences in the valence of sensory attributes between groups and to investigate possible differences in the arousal of sensory attributes between groups. These were obtained from the subjective responses to the SAM. Results showed that there were not significant differences in overall quality valence (OQ_V) (F(2,87) = 0.76, p = 0.47), overall quality arousal (OQ_A) (F(2,87) = 1.00, p = 0.37), freshness arousal (FRSH_A) (F(2,87) = 2.13, p = 0.13), flavour valence (FLV_V) (F(2,87) = 2.24, p = 0.11), flavour arousal (FLV_A) (F(2,87) = 0.64, p = 0.53), texture valence (TXT_V) (F(2,87) = 1.98, p = 0.14) or in texture arousal (TXT_A) (F(2,87) = 1.16, p = 0.32). However, the analysis of variances showed that the effect of group significantly influenced freshness valence (FRSH_V) (F(2,87) = 3.30, p = 0.04), thirst-quenching valence (THRST_V) (F(2,87) = 4.90, p = 0.01) and thirst-quenching arousal (THRST_A) (F(2,87) = 3.84, p = 0.03) so we tested multiple comparisons between groups using Tukey’s post hoc test. Results revealed that freshness valence was significantly greater in the matched identity group (M = 7.40, SD = 0.18) than in the mismatched identity group (M = 6.43, SD = 0.37), p = 0.03; thirst-quenching valence was significantly greater in the matched identity group (M = 6.67, SD = 0.25) comparatively with the mismatched identity group ((M = 5.23, SD = 0.40), p = 0.01; and thirst-quenching arousal was significantly greater in the matched identity group (M = 6.23, SD = 0.26) than in the control group (M = 4.93, SD = 0.42), p = 0.03. Therefore, as not all attributes showed significant differences, hypothesis H2 was not supported.

In order to measure the degree of association between *identity*_*score* (IDENT) and self-reported valence and arousal values and also with physiological signals values, we performed Pearson Correlations and Spearman Correlations, respectively, for the matched identity and the mismatched identity groups. For the matched identity group, we didn’t find any strong association between *identity*_*score* and the other variables—for valence: overall quality (r(30) = 0.03, p = 0.90), freshness (r(30) = 0.04, p = 0.82), thirst-quenching (r(30) = 0.40, p = 0.03), flavour (r(30) = -0.07, p = 0.72) and texture (r(30) = 0.19, p = 0.33); for arousal: overall quality (r(30) = 0.17, p = 0.37), freshness (r(30) = 0.13, p = 0.52), thirst-quenching (r(30) = 0.35, p = 0.06), flavour (r(30) = 0.08, p = 0.69) and texture (r(30) = 0.22, p = 0.23). For the mismatched identity group, we did not find any strong association between *identity*_*score* and the other variables as well—for valence: overall quality (r(30) = 0.05, p = 0.79), freshness (r(30) = 0.11, p = 0.55), thirst-quenching (r(30) = 0.16, p = 0.41), flavour (r(30) = 0.03, p = 0.86) and texture (r(30) = -0.11, p = 0.57); for arousal: overall quality (r(30) = 0.19, p = 0.32), freshness (r(30) = 0.35, p = 0.06), thirst-quenching (r(30) = 0.25, p = 0.19), flavour (r(30) = 0.16, p = 0.40) and texture (r(90) = -0.08, p = 0.68). We further the depth of analysis and performed correlations between all variables of the study for the matched and the mismatched identity groups. Results are presented in Tables 2 and 3, respectively.

**Table 2:** Correlation values among variables for the matched identity group.

	Correlations													
	1	2	3	4	5	6	7	8	9	10	11	12	13	14
1. IDENT	1.00													
2. ALPHA	-0.35	1.00												
3. HR	-0.23	0.02	1.00											
4. OQ_V	0.03	-0.20	0.09	1.00										
5. OQ_A	0.17	-0.34	0.21	0.40*	1.00									
6. FRSH_V	0.04	-0.15	0.25	0.39*	0.60***	1.00								
7. FRSH_A	0.12	-0.09	0.27	0.15	0.64***	0.70***	1.00							
8. THRST_V	0.40*	-0.37*	0.12	0.38*	0.22	-0.05	-0.05	1.00						
9. THRST_A	0.35	-0.41*	0.05	0.50**	0.51**	0.26	0.35	0.73***	1.00					
10. FLV_V	-0.07	-0.31	0.20	0.71***	0.38*	0.25	0.05	0.06	0.41*	1.00				
11. FLV_A	0.08	-0.26	0.03	0.57**	0.52**	0.12	0.06	0.18	0.49**	0.84***	1.00			
12. TXT_V	0.19	-0.43*	0.24	0.39*	0.58**	0.28	0.37*	0.48**	0.47**	0.09	0.16	1.00		
13. TXT_Ac	0.22	-0.37*	0.32	0.38*	0.48**	0.48**	0.48**	0.46*	0.65***	0.11	0.13	0.66***	1.00	
14. PRCH_INT	-0.01	-0.41*	-0.04	0.79***	0.54**	0.34	0.15	0.20	0.46*	0.83***	0.75***	0.19	0.14	1.00

* *p* < 0.05,

** *p* < 0.01,

*** *p* < 0.001.

**Table 3:** Correlation values among variables for the mismatched identity group.

	Correlations													
	1	2	3	4	5	6	7	8	9	10	11	12	13	14
1. IDENT	1.00													
2. ALPHA	0.04	1.00												
3. HR	0.02	-0.03	1.00											
4. OQ_V	0.05	-0.47**	0.07	1.00										
5. OQ_A	0.19	-0.05	-0.09	0.45*	1.00									
6. FRSH_V	0.11	-0.42*	0.08	0.81***	0.42*	1.00								
7. FRSH_A	0.35	-0.27	-0.10	0.54**	0.60**	0.75***	1.00							
8. THRST_V	0.16	-0.14	-0.16	0.72***	0.46*	0.57**	0.44*	1.00						
9. THRST_A	0.25	-0.09	-0.13	0.47**	0.60***	0.36*	0.54**	0.75***	1.00					
10. FLV_V	0.03	-0.28	0.18	0.88***	0.31	0.75***	0.42*	0.64***	0.36*	1.00				
11. FLV_A	0.16	-0.01	0.01	0.54**	0.65***	0.45*	0.42*	0.49**	0.58**	0.62***	1.00			
12. TXT_V	-0.11	-0.30	-0.07	0.62***	0.56**	0.59**	0.46*	0.58**	0.44*	0.57**	0.47**	1.00		
13. TXT_A	-0.08	-0.32	-0.14	0.45*	0.58**	0.49**	0.53**	0.52**	0.64***	0.34	0.41*	0.61***	1.00	
14. PRCH_INT	-0.03	-0.03	0.00	0.66***	0.55**	0.52**	0.37*	0.77***	0.62***	0.71***	0.64***	0.50**	0.41*	1.00

* *p* < 0.05,

** *p* < 0.01,

*** *p* < 0.001.

However, it is important to note that these tests illustrate that, for both matched and mismatched identity groups, there is an association between the reported overall quality of the beverage and the other sensory attributes, for both valence and arousal, with the strongest positive relationship between the overall quality valence and the flavour valence (for the matched identity group: r(30) = 0.71, p < 0.01, for the mismatched identity group: r(30) = 0.88, p < 0.01). Furthermore, we found that, for the matched identity group, neither EEG *α* (r(30) = -0.35, p = 0.06) or HR (r(30) = -0.23, p = 0.23) were associated with a high/low identity, and for the mismatched identity group, we also did not find any strong association between EEG *α* (r(30) = 0.04, p = 0.82) nor HR (r(30) = 0.02, p = 0.94) with the identity level.

**Fig 4:**
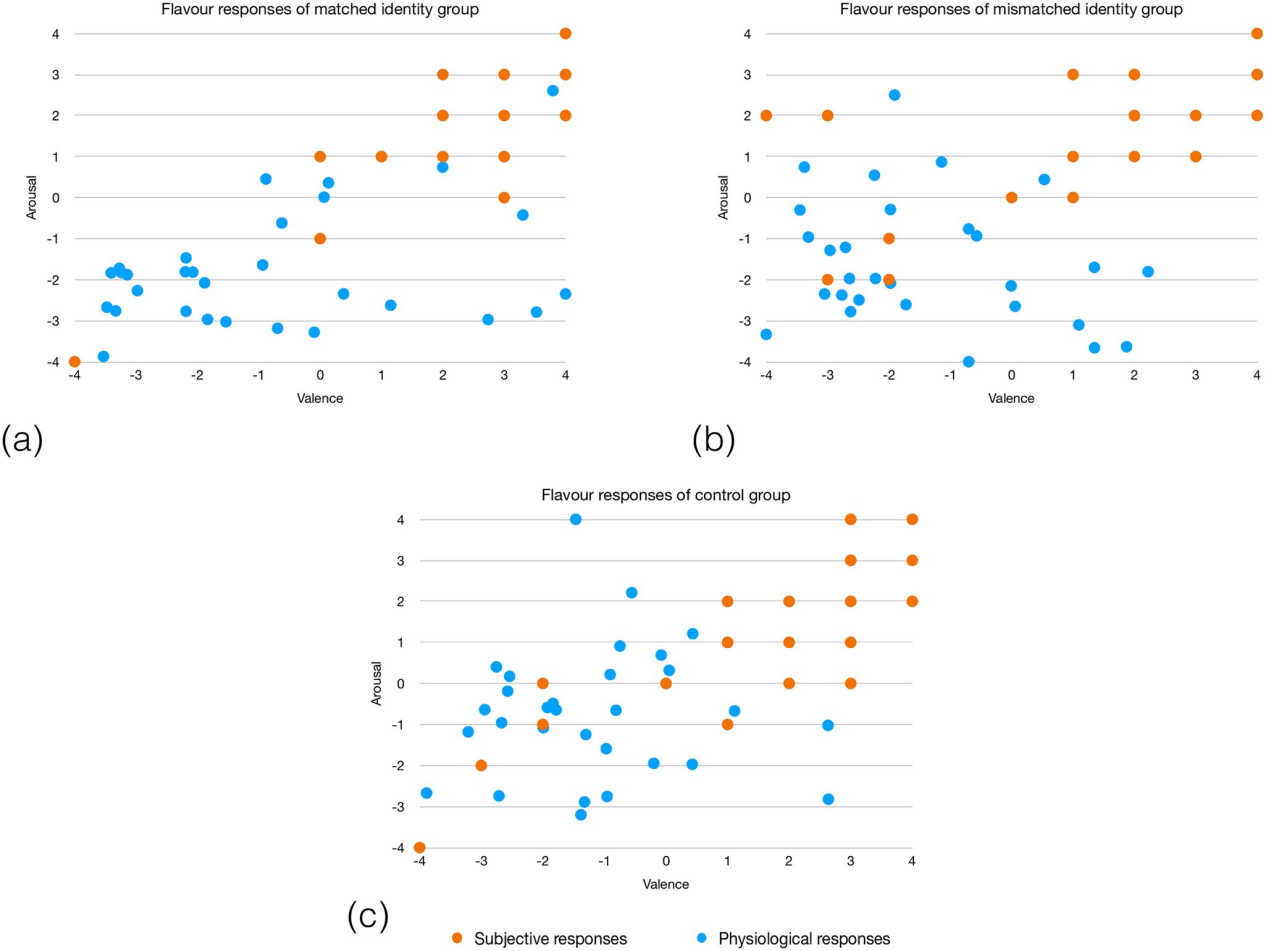
Flavour’s physiological and subjective responses of the matched identity group—Fig 4(a), the mismatched identity group—Fig 4(b) and the control group—Fig 4(c). Blue dots represent physiological responses and orange dots represent the self-reported values.

With the purpose of comparing and interpreting both physiological data and self-reported valence and arousal, we converted both physiological and subjective valence and arousal data using Min-max normalisation (Eq 2), according to previous studies [56]. For this analysis we used the raw values regarding the tasting experience (physiological and subjective). We found that, in general, participants reported a positive valence, while their physiological signals demonstrated the opposite, as presented in Fig 4 (where orange dots represent the self-reported values and the blue ones correspond to physiological responses). However, we can observe that the matched identity group presents a higher range of valence values than the mismatched identity group, having more matches of physiological and subjective responses in the right side of the valence axis, which corresponds to an emotional state of joy. We represent the differences concerning the flavour, since it is the sensory attribute more commonly evaluated through physiological signals analysis [59–61], since both EEG and BVP have been used in evaluations of taste experiences [62, 63], and since flavour is strongly associated to the overall quality of the beverage as described above.

### Purchase intentions

**Fig 5:**
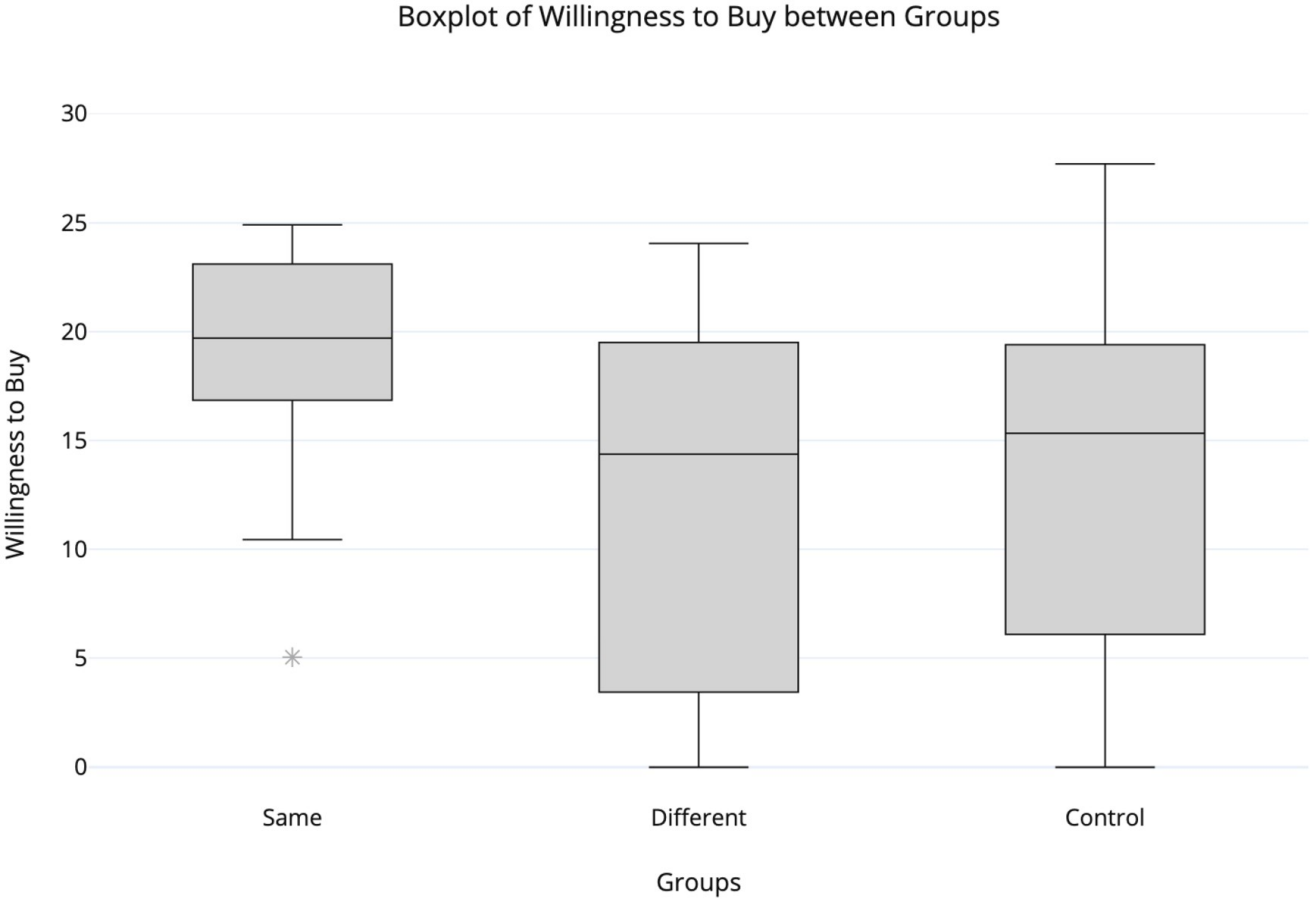
Boxplot of descriptive data pertaining to willingness to buy score between the matched identity, the mismatched identity and the control groups. Man-Whitney U tests show that the matched identity group is statistically different than the mismatched identity and the control groups for this variable. The outlier, an observation that is 1.5 x IQR greater than Q3 or less than Q1, is represented by an asterisk.

The Pearson correlation between the *identity*_*score* and the *willingness*_*to*_*buy* (PRCH_INT) did not show a significant positive association between these variables neither for the matched identity group (r(30) = -0.01, p = 0.96) or for the mismatched identity group (r(30) = -0.03, p = 0.87). However, we found significant differences between the *willingness*_*to*_*buy* score between groups (*χ*^2^ = 12.14, p < 0.01, df = 2). After computing the Kruskall- Wallis H test for testing main effects, separate Man-Whitney U tests were performed between each pair of dependent variables. As expected, according to previous studies, when performing the Man-Whitney U tests between groups, we found significant differences between the matched identity (M = 19.05) and the mismatched identity (M = 12.82) groups (*U*(*N*_*matched*_*identity*_ = 30, *N*_*mismatched*_*identity*_ = 30) = 225.50, z = -3.32, p < 0.01) and also between the matched identity and the control (M = 13.33) groups (*U*(*N*_*matched*_*identity*_ = 30, *N*_*control*_ = 30) = 270.00, z = -2.66, p < 0.01). Hence, we accepted hypothesis H3. In order to further explore whether the identification with team A or B influenced the *willingness*_*to*_*buy*, we conducted Man-Whitney U tests between subgroups. Interestingly, we found differences between AxA (M = 19.52) and AxB (M = 13.43) subgroups (*U*(*N*_*AxA*_ = 15, *N*_*AxB*_ = 15) = 65.00, z = -1.97, p = 0.05) and also between BxB (M = 18.58) and BxA (M = 12.21) subgroups (*U*(*N*_*BxB*_ = 15, *N*_*BxA*_ = 15) = 40.50, z = -2.99, p < 0.01). Fig 5 depicts the distributions and we can observe that the matched identity group has a higher median compared to the other groups. Yet, the matched identity group has a much smaller Interquartile Range (IQR), which presents further evidence that the variation was smaller compared to the mismatched identity and the control groups as already demonstrated in the Mann-Whitney analysis. We represented these data in boxplots, since they are more visually informative, showing the minimum and maximum value of *willingness*_*to*_*buy* for each group, represented by the box whiskers. Moreover, Tables 2 and 3 show a significant strong correlation between *willingness*_*to*_*buy* and the beverage’s flavour (valence), for both matched (r(30) = 0.83, p < 0.01) and mismatched identity (r(30) = 0.71, p < 0.01) groups.

## Discussion

Here, we explored the influence of ingroup/outgroup identification on the sensory perception of a beverage that sponsors the ingroup/ougroup team (at both neurophysiological and subjective levels) and on the purchase intention of that beverage. For this purpose, our study protocol involved tasting a beverage and the subsequent response to a questionnaire about beverage’s sensory attributes as well as its purchase intentions, while physiological signals (EEG and BVP) were being collected.

We expected that participants identified with the team who tasted the ingroup/outgroup beverage would present positive/negative physiological responses (positive valence) as well as positive/negative subjective responses. Moreover, we expected that participants with a high level of identification with a team would have greater self-reported and physiological arousal values. Interestingly, our results showed that neither physiological nor subjective responses showed significant differences between groups (matched identity, mismatched identity and control). Therefore, H1 and H2 were not supported. However, we found that despite the absence of differences, participants reported to enjoy the beverage, while their physiological signals demonstrated the opposite. Furthermore, and contrary to the assumption driven by the literature, our results indicate that the high/low level of identity was not associated with more positive/negative self-reported responses nor with neuronal responses. Moreover, the high/low level of identity was also not associated with greater/lower arousal values. This suggests that sensorial experience might result from the Top-Down mechanisms, since it seems that the sensory perception is driven by cognition, which may have varied according to several factors, such as prior experience, knowledge, and one’s personal expectations and emotional state [64]. Regarding the purchase intentions, our study revealed that the level of team identification in itself did not lead to a greater willingness to buy. However, independently of how much one feels identified to one group, our results demonstrate that the group a participant is identified with has an effect on his/her purchase intentions. More specifically, participants who tasted the ingroup beverage (matched identity group) are more likely to buy the beverage compared to the others who tasted the outgroup beverage (mismatched identity group) and also to the ones from the control group, which means that the group to which a participant identifies with influences his/her purchase intentions of the sponsor’s product.

According to the social identity theory, a positive social identity is derived from favourable comparisons between ingroups and outgroups. Thus, we expected that people from the ingroup would have higher purchase intentions of their team’s sponsor product when comparing to the outgroup. Surprisingly, and contrary to previous research [24], our data showed that being from the ingroup/outgroup does not influence the physiological responses (Bottom-up mechanism) or even the subjective responses given through the questionnaire about the sensory attributes (Top-down mechanism). The concept of social identity complexity may subtend these results wherein it is evident that a positive attitude can be directed toward the outgroup, given that individuals may have overlapping group identities [65], which can vary according to diverse factors (personal needs and values and situational factors, such as stress and ingroup threat). In fact, the pandemic situation that prevented fans from attend football games during the period of data collection may have contributed to behavioural and attitudinal variations in relation to prior studies, since researchers found that perceived event quality and team performance influences fans’ intentions to purchase a sponsor’s product [66]. Nevertheless, having the data normalised, we found that apart from being from the ingroup/outgroup, almost all subjects reported high valence and arousal values while physiological signals demonstrated low values of valence. This means that people reported to be happy/excited about trying the beverage while the physiological signals showed that participants were feeling sad/depressed/angry [67], which is contrary to previous studies indicating a strong correlation between peripheral physiological measures (e.g., BVP) and the self-reported answers using SAM [35–37] was verified. Indeed, these results demonstrate that, although a higher identification with team A or B does not influence the self-reported subjective experiences or neuronal responses, the presented mechanism seems to be a Top-Down mechanism, since participants felt the necessity to report a positive experience in tandem with physiological signals demonstrating the opposite. Thus, one might assume that there is an heuristic that led to a cognitive bias when reporting the sensorial experience [64, 68]. This can also be explained by the tendency of people to report an answer in a way they thought to be more socially acceptable than what they truly felt in the moment (social desirability), which is a common problem caused by self-reported answers [69]. Moreover, we found that physiological signals from the matched identity group had more matches on the right side of the axis (high positive valence values), than the mismatched identity group. This highlights that, in fact, a Bottom-up mechanism, in which one’s identification influences the sensorial experience of the beverage, can also be present.

Crucially, we found an absence of association between the level of identification and both self-reported and physiological responses. Moreover, we also discovered that a higher level of identification with a team does not influence willingness to buy the sponsor’s products (i.e., it is enough that the brand represents that team). This finding aligns with a phenomena called “CORFing”, in which the closer the identification to the team and degree of commitment by the fans, the greater the risk the fan has of suffering a loss in self-esteem if their team has lost [70]. However, this work also corroborates results from previous studies [2, 9], given that the purchase intentions were found to be greater in participants experiencing ingroup beverages and so, hypothesis H3 was supported. This demonstrates that it is not the sensory attributes of the beverage that led to a more positive willingness to buy, but instead it was the proper brand (sponsoring brand of the participant’s team) that influenced consumer intentions, which concurs with previous findings [71]. Furthermore, we found that the flavour of the beverage was strongly associated with its purchase intentions, for both matched and mismatched identity groups. This means that one could might report and appreciation of both ingroup and outgroup sponsoring beverages, however their team identification would lead them to buy the sponsoring beverage from their own team, as this is way to acknowledge the sponsors for supporting their team [2, 8]. This finding can also be explained by the fact that fans’ goodwill toward their team is often transferred to the sponsors’ products [6]. The fact that the majority of the participants of this study were young (aged between 18 and 25) might underlie these findings, since other have verified that younger consumers tend to be more receptive to sponsorship [72].

Previous studies have demonstrated one’s social identity influences consumer intentions towards branded products [2, 9, 71]. Since sponsors make great investments in sports, it is crucial to understand if their effort is rewarded. The methodology employed in the majority of the studies concerning the sponsorship effect consisted in questionnaires. Furthermore, it is extremely hard to isolate the sponsorship effect because brands invest in several marketing strategies at the same time. As such, results from this study add to extant literature by allowing a better comprehension of people’s reaction to sponsored brands and how it likely affects their future behaviours. Moreover, this study highlights that team identification seem to be more important than the quality of the sponsor’s product.

Early research demonstrated that beverage’s labels, but not the taste, influenced beverage’s rating and preference [73]. More recently, another study tested the effects of brand cues in gustatory processing of the same beverage and showed that people preferred beverages associated with the famous brands over the others, despite the beverage being the same on all the brands’ packages [74]. Furthermore, this study not only illustrated the strong effects of brand cues on self-reported pleasantness but also on neural responses, since areas of the brain involved in encoding reward value were activated more by strong brand cues than the others lesser known brands. Despite the importance of those studies, people’s social identity was overlooked. Hence, our study provides innovative results, since we not only show the dissociation of physiological responses (which were collected synchronously) with the self-reported ones by subjects while tasting the same beverage, but we also investigate the influence of ingroup and outgroup on those future behaviours.

In the future, larger samples could be collected in order to further enrich the understanding of how consumers react to sponsor products. Also, the number of female participants was not matched to the number of male participants since there was a greater accessibility to male fans compared with female ones. Although this is not a limitation of our study because it translates a real proportion of male/female football fans in Portugal, future studies might consider increasing the testing of female fans since studies have shown gender has been suggested to influence purchase decision-making [75] and it would allow further comparisons. Finally, it would be interesting to repeat this study in a post-pandemic scenario (i.e., live football games with fans in the stadiums), since game attendance is a sign of behavioural loyalty [76] that has been suggested to reflect positively on fan reactions to sponsors [2, 77].

## Conclusion

Here we investigate the impact of team identification on the sensorial experience of tasting a beverage sponsoring by the ingroup or the outgroup football sports team. Although much research has investigated purchase decisions in sponsoring brands, here we attempt to assess how these decisions might be mediated by the sensorial experience of the sponsoring product itself as assessed by EEG and BVP and subjective self-reports concerning sensory attributes of the beverage. Considering the literature concerning the effect of sponsors on fans, we predicted that team identification would modulate the proper sensorial experience (experience that one undergoes including flavour, freshness, thirst-quenching and texture when tasting the beverage), i.e. a beverage that sponsors one’s team would taste better. However, no participant seemed to enjoy the beverage (as given by physiological data), independently of their ingroup or outgroup identification. Surprisingly, however, almost all participants reported enjoying the beverage in spite of the negative affect given by the physiological signals. Nonetheless, participants who identified with the football team sponsored by the beverage were willing to pay more for that beverage. Thus, our study emphasizes the overwhelming influence of the brand on consumer decisions, outplaying the importance of product quality.

## Supporting information

S1 Appendix Research questions. Includes questions regarding team identification, subjective sensorial experience and purchase intentions.
(PDF)
