# Supplementary material for: Sports ingroup love does not make me like the sponsor’s beverage but gets me buying it
Source: PLoS One. 2021 Jul 28;16(7):e0254940. doi: 10.1371/journal.pone.0254940 (PMC8318299; doi:10.1371/journal.pone.0254940)
Supplement: S1 Appendix — Includes questions regarding team identification, subjective sensorial experience and purchase intentions. (PDF) [file pone.0254940.s001.pdf]

Questions concerning **team identification**:

1. How much are you interested in football?
2. Do you support any football team? Which one?
3. Do you own a membership card? From each team?
4. How much do you identify with team A?
5. How much do you oppose team A?
6. Before the COVID-19 pandemic situation, how often did you used to watch a team A's matches in a stadium?
7. How much do you identify with team B?
8. How much do you oppose team B?
9. Before the COVID-19 pandemic situation, how often did you used to watch a team B's matches in a stadium?

Questions concerning **subjective sensory experience**:

1. How pleasant was your experience concerning the overall perceived quality of the beverage?

Extremely Unpleasant

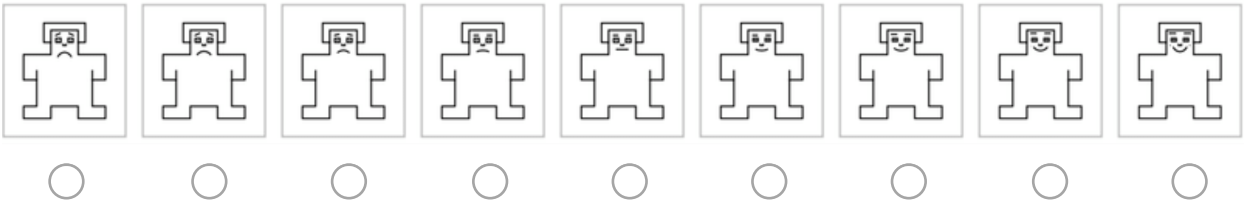

Extremely Pleasant

2. How intense was your experience concerning the overall perceived quality of the beverage?

Extremely relaxing

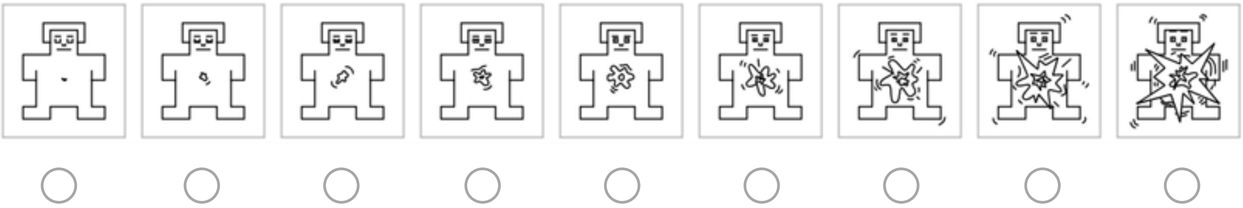

Extremely exciting

3. How pleasant was your experience concerning the sensory attribute of being refreshing?
4. How intense was your experience concerning the sensory attribute of being refreshing?

5. How pleasant was your experience concerning the sensory attribute of being thirst-quenching?
6. How intense was your experience concerning the sensory attribute of being thirst-quenching?
7. How pleasant was your experience concerning the flavour of the beverage?
8. How intense was your experience concerning the flavour of the beverage?
9. How pleasant was your experience concerning the texture of the beverage?
10. How intense was your experience concerning the texture of the beverage?

Questions concerning **purchase intentions**:

1. I would recommend this beverage to my family/friends.
2. I would like to buy this beverage when it becomes available.
3. How much would you be willing to pay for this beverage?
    - ◯ I would not be willing to pay for this drink at all.
    - ◯ Up to 2€.
    - ◯ More than 2€.
